# Pedestrian Flow Model Based on Cellular Automata Under Visual Trajectory and Multi-Scenario Evacuation Simulation Research

**DOI:** 10.3390/s26051405

**Published:** 2026-02-24

**Authors:** Yueyue Chen, Jinbao Yao, Chenze Gao, Haoyuan Guo

**Affiliations:** School of Civil Engineering, Beijing Jiaotong University, Beijing 100044, China

**Keywords:** pedestrian flow analysis, YOLO-DeepSORT, cellular automata, evacuation simulation, panic behavior

## Abstract

Precise modeling and simulation of pedestrian flow are crucial for public space safety design and emergency management. This study proposes an interdisciplinary method integrating computer vision and cellular automata (CA). First, unidirectional pedestrian flow video data with different densities were collected from an overpass scene via controlled experiments. High-precision pedestrian trajectory extraction and tracking were achieved using the YOLO 11 model and DeepSORT algorithm, with image distortion corrected by perspective transformation. For the first time, the probability distribution of pedestrian turning angles derived from trajectory analysis was converted into data-driven transition probabilities for the Moore neighborhood in the CA model. An improved evacuation model was then constructed, comprehensively considering real-data-based transition probabilities, speed–density distribution, panic coefficient, individual life value, and hazard source dynamics. Multi-scenario simulations show that moderate panic may shorten evacuation time, while excessive panic causes behavioral disorders; group movement is constrained by the slowest individual, and increased hazard source speed reduces the proportion of safe pedestrians. This study provides new insights and methodological support for refined pedestrian evacuation simulation and safety management.

## 1. Introduction

Pedestrian flow mechanics and emergency evacuation simulation, as key research fields in crowd safety management and public building optimization design, have long received extensive attention from multiple disciplines such as traffic engineering, safety science, and computer simulation. Against the backdrop of the accelerating urbanization process, the gathering of people in large public spaces such as subway stations, overpasses, and sports venues has become increasingly common. As a result, safety hazards such as congestion, stampedes, and low evacuation efficiency have become more prominent. Therefore, a thorough understanding of the fundamental laws of pedestrian movement and the development of high-precision and highly reliable pedestrian simulation models based on this are of crucial theoretical significance and practical value for formulating scientific emergency management strategies, optimizing the spatial layout of buildings, and ensuring public life safety.

At present, pedestrian flow research mainly relies on two types of methods: empirical observation and computer simulation.

In terms of empirical research, extracting pedestrian trajectory data through video recordings has become the mainstream method. With the rapid development of computer vision technology, especially the maturity of object detection based on deep learning, such as the YOLO series models [1,2], and multi-object tracking algorithms, such as DeepSORT [3], it has become possible to automatically and precisely obtain massive pedestrian trajectory data from complex scenes [4]. In particular, the Vision Transformer (ViT) architecture was successfully applied to instance segmentation in dense crowds in 2024, significantly enhancing the tracking robustness under complex occlusions [5]. In recent years, correlation methods such as ByteTrack [6] have further enhanced tracking stability in dense scenarios, and the introduction of the Transformer architecture [7] has also provided a new paradigm for end-to-end multi-object tracking. These data provide a solid foundation for analyzing the micro-movement characteristics of pedestrians. However, most vision-based studies focus solely on trajectory extraction and statistical analysis, lacking effective integration with downstream simulation models—resulting in isolated data that fail to directly inform behavioral rule calibration.

In terms of simulation modeling, the Cellular Automata (CA) model has been widely applied in pedestrian evacuation simulation due to its characteristics such as clear rules, high computational efficiency, and ease of simulating emergent behaviors of complex systems [8,9]. However, traditional CA models often rely on simplified, hypothetical behavior rules, and their model parameters are often not adequately calibrated with real pedestrian behavior data, resulting in deviations between the simulation results and real scenarios [10,11]. In response to this limitation, recent research trends emphasize data-driven, i.e., using real trajectory data to calibrate and optimize model parameters [12], or introducing machine learning methods to enhance model decision-making capabilities [13]. For example, spatio-temporal interaction between pedestrians is learned through graph neural networks and deep reinforcement learning, and the local evolution rules of CA models are dynamically predicted, achieving more realistic results when simulating panic evacuation [14,15]. Despite significant progress, existing research still faces unresolved challenges that hinder the accuracy and applicability of evacuation simulations: Pedestrian movement in emergencies is jointly determined by microscopic individual characteristics, mesoscopic group interactions, and macroscopic environmental dynamics. Existing models rarely integrate these three dimensions into a unified framework, leading to incomplete simulation of evacuation dynamics.

There are still two core issues that need to be urgently addressed: First, there is a certain degree of disconnection between vision-based behavior analysis research and CA-based simulation modeling research. The underlying behavioral rules of many simulation models lack direct drive and verification from real trajectory data. Secondly, when simulating emergency evacuation scenarios, the model’s handling of social and psychological factors such as pedestrian panic is often rather simplistic, failing to fully depict the dynamic evolution of panic emotions and their nonlinear impact on pedestrian decision-making and movement speed.

In order to fill the above research gaps, this paper conducts a cross-study integrating computer vision and cellular automata. In this study, the one-way pedestrian flow video data under different density conditions were systematically collected. Subsequently, the advanced YOLO 11 object detection algorithm and DeepSORT tracking algorithm were used to automatically extract and generate high-precision pedestrian movement trajectories. By performing perspective transformation correction and statistical analysis of the trajectory data, we quantify and extract the steering angle probability distribution of pedestrian motion from the empirical data for the first time, and transform it into the data-driven transfer probability of the Moore neighborhood in the cellular automaton model.

This paper constructs an improved cellular automaton evacuation model. This model not only introduces the transfer probability based on real data, but also comprehensively considers the dynamic influence of multiple factors such as the pedestrian density–speed distribution, panic coefficient, individual health value, and the diffusion speed of hazard sources on the pedestrian movement speed. Thus, it can more realistically simulate the evacuation behavior and group dynamic process of pedestrians in emergency scenarios such as fire and armed robbery.

**Figure 1:**
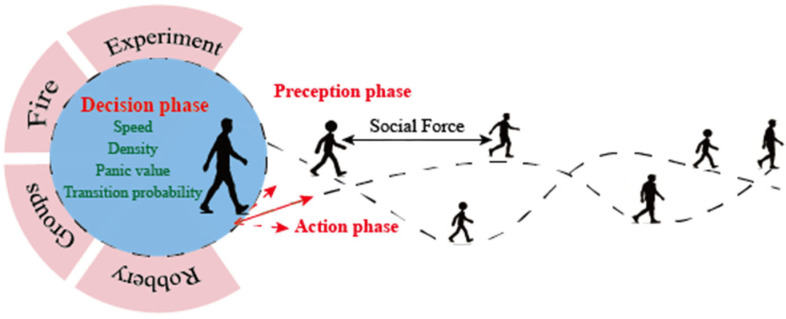
Framework diagram.

The overall research framework is illustrated in Figure 1.

## 2. Experiment

**Figure 2:**
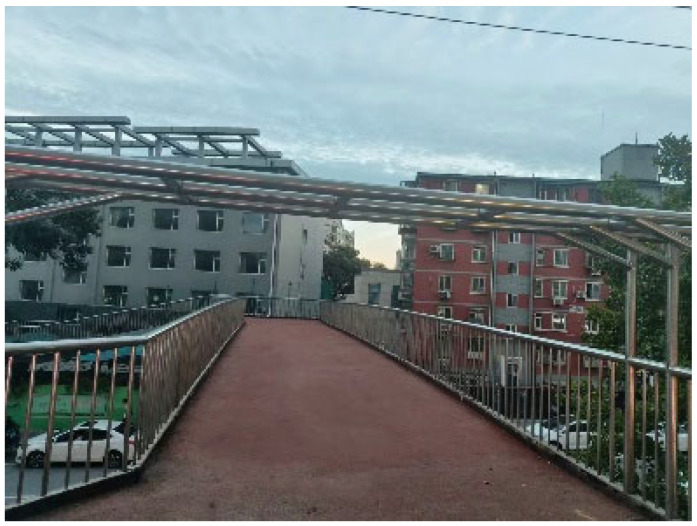
Test site.

The test site was selected at the Jiaotong University Road overpass. The bridge deck is shown in Figure 2. This bridge meets the general standards of urban overpasses. The bridge deck is 17.6 m long and 4.3 m wide. There are entrances and exits at both ends of the overpass, simulating a real pedestrian crossing scene.

Twenty-five volunteers (21 males and 4 females) were recruited to voluntarily participate in the experiment, with their ages ranging from 18 to 35. Although the participants knew the general information of the project, they were unaware of the specific measurement details and the content of the analysis. In each experiment, participants were positioned at the entrance of the overpass (eastern side) and instructed to walk naturally in a single direction toward the exit (western side) at their own preferred speed, without overtaking or interfering with others unless necessary. No specific pacing or path guidance was provided, ensuring that the collected data reflected natural walking behavior under controlled spatial constraints. Tests were conducted in sequence according to the working conditions of 5 people, 10 people, 15 people, and 20 people. During each group of experiments, the walking videos of pedestrians were recorded throughout the process to ensure that the walking paths and trajectories of each person could be clearly recorded.

The camera model used is TP-LINK, with a focal length of 12 mm, a display resolution of 2560 pixels (width) × 1440 pixels (height), and a frame rate of 25 frames per second (fps). Only the data from the bridge deck measurement area were collected.

The experimental environment for pedestrian target tracking is Windows 10 operating system, Python 3.9, 16.0 G RAM, Intel (R) Core (TM) i7-9750U processor and NVIDIA GeForce GTX 1660 Ti (6 GB) GPU.

To study the influence of pedestrian density on unidirectional walking speed, trajectory distribution and congestion effect, this paper designs four sets of experimental conditions with different pedestrian scales, as shown in Table 1, to simulate unidirectional evacuation scenarios ranging from low density to medium-high density.

## 3. Pedestrian Flow Analysis Based on YOLO 11+DeepSORT

### 3.1. Pedestrian Trajectory

Extract the trajectory data of pedestrians from the video through YOLO 11 and DeepSORT. Trajectory extraction employs the YOLO11 detector and the DeepSORT tracker. YOLO11 uses the default parameter configuration, and the input images maintain their original resolution. The DeepSORT tracker predicts the target position through Kalman filtering and performs data association based on appearance features (extracted by WideResNet). Abnormal trajectory points are filtered by distance thresholds and smoothed using a moving average with a window size.

**Figure 3:**
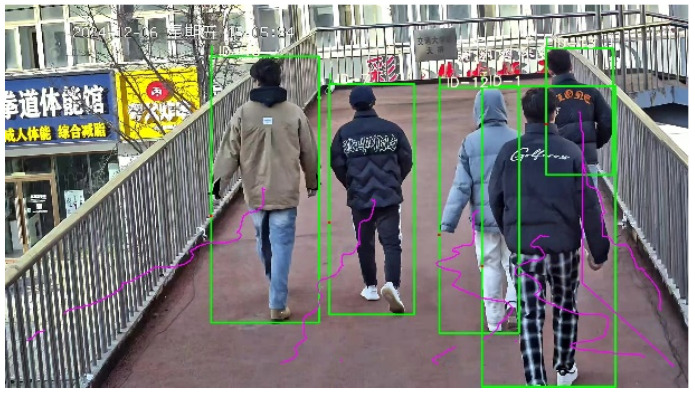
Experimental diagram of a 5-person group.

**Figure 4:**
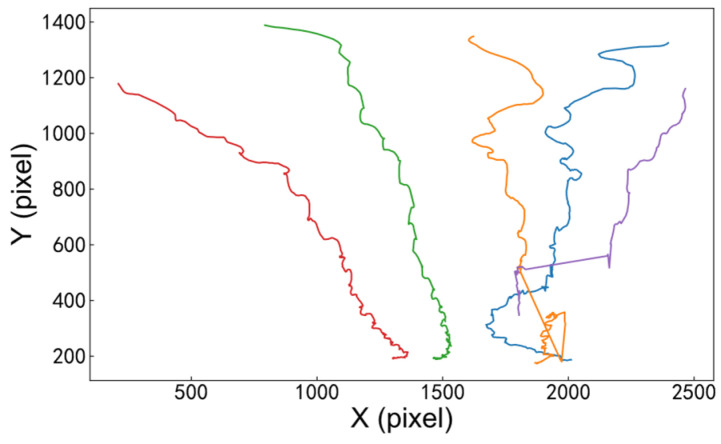
Motion trajectory (*N* = 5).

The trajectory of each pedestrian can be represented as a series of coordinate points (x, y), where x and y respectively represent the position of the pedestrian on the plane of the overpass. The horizontal and vertical coordinates respectively represent the camera recognition pixels. Figure 3 shows the scene of trajectory recognition, and Figure 4 shows the trajectory recognition results with pixels as the horizontal and vertical coordinates.

As the cameras are placed at the bridgehead, the pedestrian trajectories in the image show a perspective effect where the closer ones appear larger and the farther ones smaller. The pedestrian trajectories in the foreground are relatively sparse, while those in the distance are more compact. The perspective transformation matrix can be calculated by comparing the points in the provided image with their corresponding actual coordinates.

The application of perspective transformation matrices in code is based on the principle of planar projection transformation in computer vision. Its core is to map the pixel coordinates in the image to the physical coordinates in the real world.

The perspective transformation is represented by a 3 × 3 homomorphic matrix *H*, as shown in Equation (1):

(1) $$\left[\begin{array}{c} x^{\prime }\\ y^{\prime }\\ w^{\prime } \end{array}\right]=H\left[\begin{array}{c} x\\ y\\ 1 \end{array}\right]=\left[\begin{array}{ccc} h_{11} & h_{12} & h_{13}\\ h_{21} & h_{22} & h_{23}\\ h_{31} & h_{32} & h_{33} \end{array}\right]\cdot \left[\begin{array}{c} x\\ y\\ 1 \end{array}\right]$$

Normalization gives the true coordinates:

(2) $$x^{\prime \prime }=\frac{x^{\prime }}{w^{\prime }},y^{\prime \prime }=\frac{y^{\prime }}{w^{\prime }}$$

where $\left(x,y\right)$ represent the pixel coordinates of the image; $\left(x^{\prime },y^{\prime }\right)$ represent the transformed unnormalized coordinates, including the original calculation results of the perspective distortion effect; *w*′ is the scaling factor of homogeneous coordinates; $\left(x^{\prime \prime },y^{\prime \prime }\right)$ are the corrected actual physical coordinates.

The perspective transformation matrix H is calculated through four pairs of control points. The source points are the image pixel coordinates of the four ground markers on the bridge surface ([2071, 384], [2334, 805], [765, 864], [1104, 449]), and the target points are the measured physical coordinates ([17.6, 0], [8.8, 0], [8.8, 4], [17.6, 4], unit: m). The homography matrix is solved using the findHomography function of OpenCV based on the least squares method. The re-projection error analysis shows that the average positioning error of the control points is 0.127 m, which is less than the cell size of 0.4 m and can be ignored for the subsequent analysis.

Solve the overdetermined equations by the least square method to find the optimal *H* matrix. By selecting the source point and the actual target point in the video, the perspective transformation matrix can be obtained. Equation (3) is as follows:

(3) $$H=\left[\begin{array}{ccc} 0.008 & -0.086 & 217.940\\ -0.043 & 0.027 & 77.838\\ 0.002 & 0.017 & 1 \end{array}\right]$$

**Figure 5:**
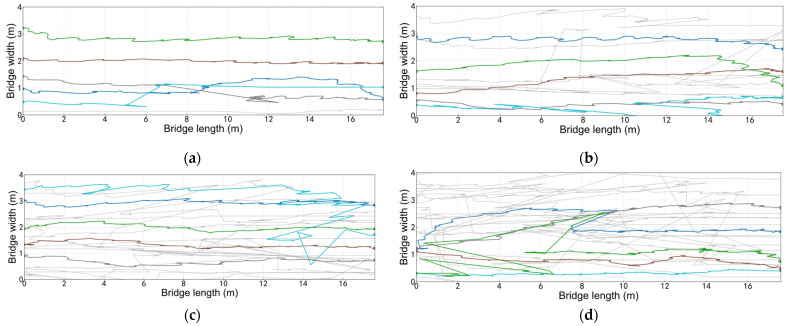
Pedestrian trajectory map (color is the random extraction of 5 people’s path, dark color is the rest of the pedestrian tracks). (**a**) *N* = 5; (**b**) *N* = 10; (**c**) *N* = 15; (**d**) *N* = 20.

The pedestrian trajectory map adjusted by the perspective transformation moment is rather messy. To clearly present the overall distribution and local characteristics of pedestrian trajectories under different population sizes, Figure 5 uniformly visualizes the trajectory data from each experiment: the trajectories of 5 randomly selected pedestrians are highlighted in colored lines, while the trajectories of the remaining pedestrians are presented in uniform light gray lines.

The trajectory graph in Figure 5 shows that when the bridge deck is not too crowded, the pedestrian density is [0.071, 0.286] ped/m^2^. Pedestrians do not significantly change their walking routes. The vertical coordinate of the trajectory curve is messy within [0, 1], indicating that pedestrians walk on the right side in a complex and interlaced manner with a large number of people, and the movement of pedestrians is often not completely regular.

Because pedestrians may suddenly change direction, speed, or perform some irregular actions, such as avoiding obstacles or interacting with others, these irregular movement behaviors may cause errors in trajectory recognition algorithms based on conventional motion patterns, causing the trajectory points to deviate from their actual positions.

### 3.2. Steering Angle Distribution

For each trajectory, calculate the steering angle $\theta_{t}$ with three consecutive points $\left(x_{t-1},y_{t-1}\right),\left(x_{t},y_{t}\right),\left(x_{t+1},y_{t+1}\right)$:

Moving forward:

(4) $${\vec{v}}_{1}=\left(x_{t}-x_{t-1},y_{t}-y_{t-1}\right)$$

The next step:

(5) $${\vec{v}}_{2}=\left(x_{t+1}-x_{t},y_{t+1}-y_{t}\right)$$

The steering angle can be expressed by Equation (6):

(6) $$\theta_{t}=\mathrm{arc}\cos\left(\frac{{\vec{v}}_{1}\cdot {\vec{v}}_{2}}{\left\|{\vec{v}}_{1}\right\|\cdot \left\|{\vec{v}}_{2}\right\|}\right)\cdot sign\left(cross\left({\vec{v}}_{1},{\vec{v}}_{2}\right)\right)$$

where the sign of the cross product indicates a left turn (positive) or right turn (negative).

**Figure 6:**
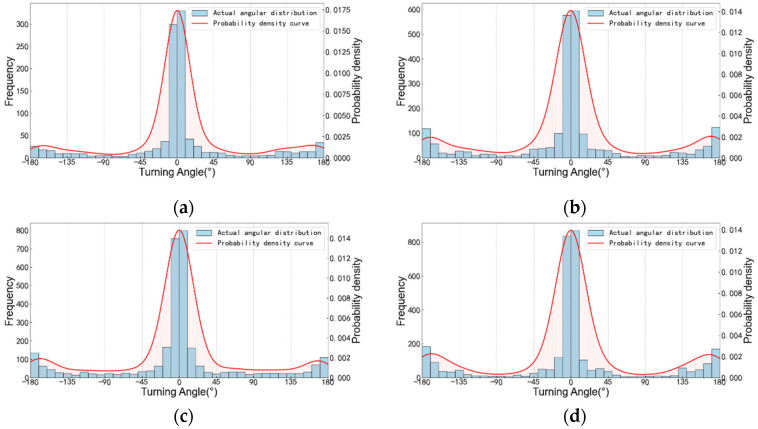
Steering angle distribution map. (**a**) *N* = 5; (**b**) *N* = 10; (**c**) *N* = 15; (**d**) *N* = 20.

Based on the pedestrian trajectory obtained from the video analysis, the Gaussian kernel density estimation can be used to calculate the probability density curve of the pedestrian’s turning angle, as shown in Figure 6:

In Figure 6, the probability density curve reaches its peak near 0°, indicating that pedestrians tend to walk straight most of the time, which is consistent with the conclusion of Section 3.1. Their movement is highly continuous, and turning behavior is relatively rare. Especially when the number of people is smaller, this situation becomes more obvious.

The distribution curve is basically symmetrical on both sides of 0°, indicating that pedestrians have no significant preference for left and right turns. The probability densities of left and right turns are similar. Based on the probability density curve, the probability values of each turning interval can be integrated. This symmetry may indicate that there are no obvious directional restrictions or biased designs in the environment, which is consistent with the design situation of this working condition.

The straight-line-dominated, left–right symmetrical distribution and discrete peaks of steering angles provide an important basis for pedestrian movement modeling, environmental optimization or abnormal behavior detection.

### 3.3. Performance Evaluation

Confidence level plays a key role in the object detection process of YOLO. By setting a confidence threshold, the test results with low confidence can be filtered out and only those with high confidence can be retained. Among multiple overlapping bounding boxes, select the one with the highest confidence level and suppress the others with lower confidence levels. Confidence level can be used to evaluate the detection performance of a model in different scenarios by, for example, drawing a confidence level curve or calculating the average confidence level.

**Figure 7:**
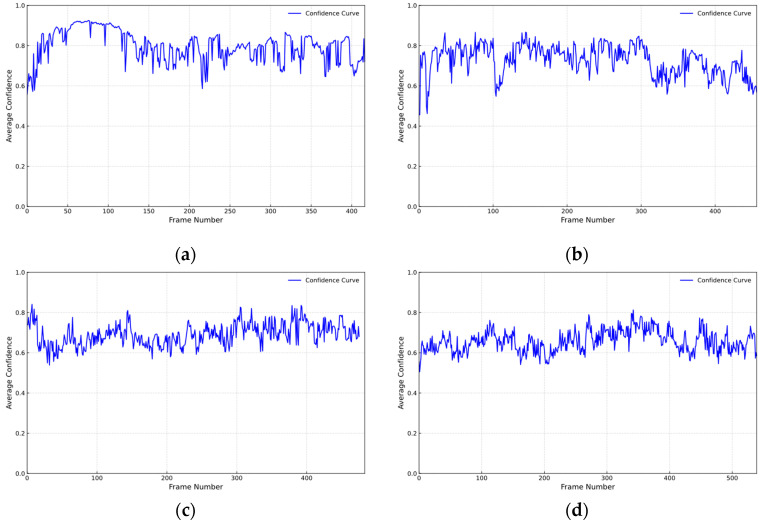
Confidence level. (**a**) *N* = 5; (**b**) *N* = 10; (**c**) *N* = 15; (**d**) *N* = 20.

Figure 7 shows the change of the recognition confidence of the YOLO object detection model for each frame in the video sequence.

Figure 7 shows that the confidence level fluctuates throughout the entire video sequence and decreases as the number of people increases. It can be seen that the pedestrian density increases, and pedestrian occlusion affects the target tracking state. However, the confidence level remains stable at [0.6, 0.8], indicating that the targets in these frames of the video are clear, and the detection performance of the model is relatively high, capable of reliably detecting the targets.

## 4. Pedestrian Evacuation Simulation Based on Cellular Automata

Cellular automata simulate complex global behaviors through simple local rules and are particularly suitable for modeling spatial explicit processes such as pedestrian flow. A typical CA model consists of four basic elements: $\left(L,S,N,f\right)$, where *L* represents the regularly divided cellular space and, in this study, the discretized two-dimensional grid of the bridge deck. *S* is a finite set of states, *N* defines the domain, and *f* is a local evolution rule that determines the state of the cell at the next moment.

**Figure 8:**
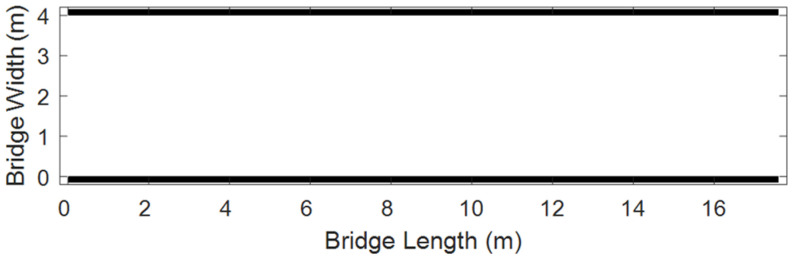
Bridge deck simulation.

The physical space where pedestrians are located (such as the bridge deck of a sky bridge) is discretized into a regular two-dimensional grid, and each grid cell is called a “cell”. The site simulation is shown in Figure 8:

At any given moment, each cell can only be in one of two states: “idle” or “occupied” by a pedestrian. The movement of pedestrians is defined by the neighborhood of the cell in which they are located. This paper adopts the most widely used Moore neighborhood, that is, the neighborhood of a cell is composed of eight adjacent cells, namely, four orthogonal directions of front, back, left and right, and four diagonal directions of left front, left back, right front and right back. That is, $N_{\left(i,j\right)}$ can be expressed as Equation (7):

(7) $$N_{\left(i,j\right)}=\left(i+\Delta x,j+\Delta y\right)|\Delta x,\Delta y\in \left\{-1,0,1\right\}$$

At each simulation time step, the pedestrian will, based on the preset evolution rules and the Moore neighborhood state of the current cell they are in, decide which adjacent cell to move to next. That is, the state of the central cell (*i*,*j*) at time *t* + 1 is jointly determined by its own state and that of its neighboring cells at time *t*. The evolution rules are as follows:

(8) $$S_{\left(i,j\right)}\left(t+1\right)=f\left(S_{\left(i.j\right)}\left(t\right),S_{N_{\left(i,j\right)}}\left(t\right)\right)$$

Traditional CA models typically assign empirical or equal probabilities to these moving directions. One of the core innovations of this study lies in using the statistical distribution of pedestrian turning angles obtained from the aforementioned visual trajectory analysis to directly and objectively calibrate the data-driven transfer probabilities of each direction in the Moore neighborhood. Thus, the microscopic characteristics of real pedestrian movement are seamlessly embedded into the simulation rules, significantly enhancing the authenticity and credibility of the model.

### 4.1. Moore Neighborhood Transfer Probability

**Figure 9:**
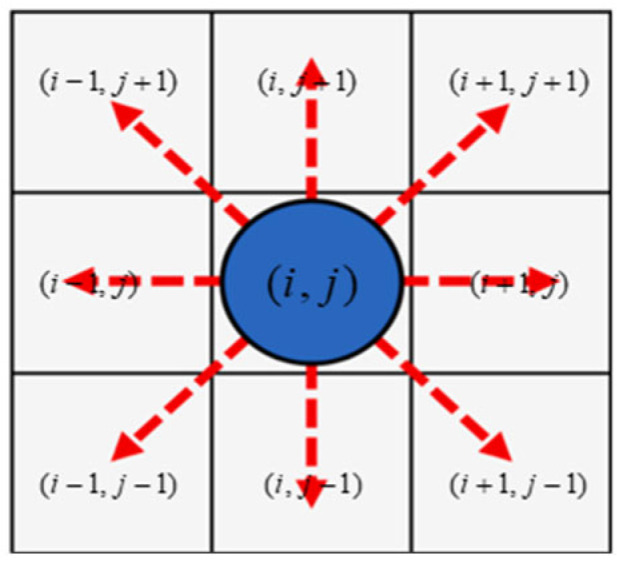
Moore neighborhood.

**Figure 10:**
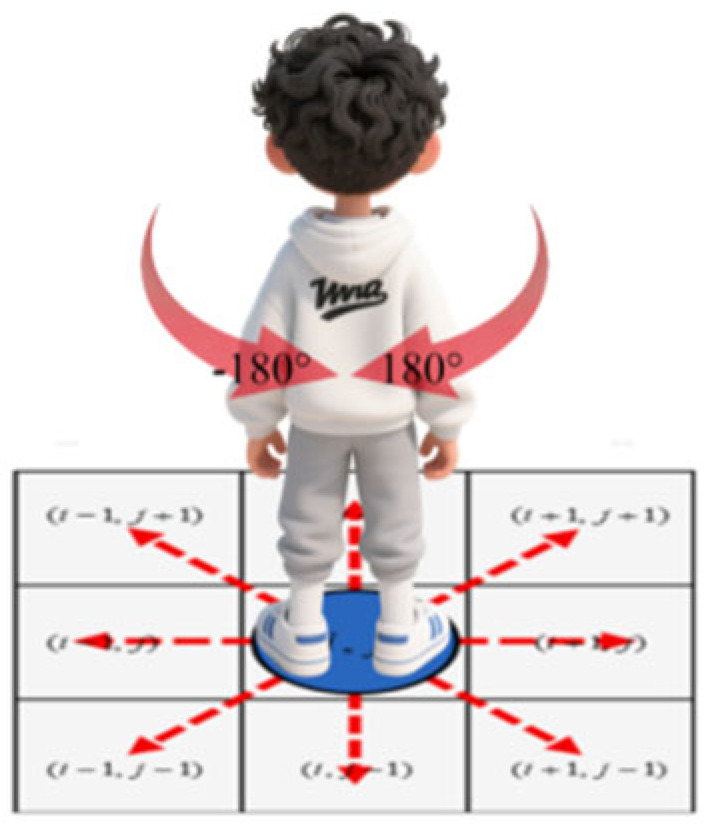
Moore neighborhood and steering angle comparison.

To effectively embed the quantitative characteristics of pedestrian turning behavior obtained based on real trajectory analysis into the evolution rules of cellular automata, it is necessary to establish a mapping mechanism from the continuous movement direction to the discrete grid movement. Figure 9 visually presents the classic Moore neighborhood structure in cellular automata. Figure 10 further establishes the spatial geometric correspondence between the Moore neighborhood and the actual turning angle of pedestrians. It divides and maps the continuous turning angle intervals (such as −180° to 180°) to eight specific discrete directions within the Moore neighborhood. Through this key mapping, we were able to obtain the probability of the continuous steering angle from the empirical analysis, as shown in Table 2.

By identifying the statistical probability density of the turning angle, the transition probabilities of each cell in the Moore neighborhood can be integrated. By calculating the average value, the general applicable probability was obtained, and the results are shown in Table 2.

### 4.2. Incorporating Behavioral and Environmental Factors

Pedestrian traffic is very complex and is influenced by various internal and external factors. Internal factors include the pedestrian’s age, gender, health condition, psychological factors, relevant knowledge and experience, etc. External factors include the structure and layout of the built environment, social relations, and interaction with other pedestrians around. In the process of simulating pedestrian traffic in this paper, factors such as the density–speed relationship of pedestrians and the influence of panic were considered, and multiple scenarios were simulated and the traffic characteristics were analyzed.

#### 4.2.1. Velocity–Density Distribution

**Figure 11:**
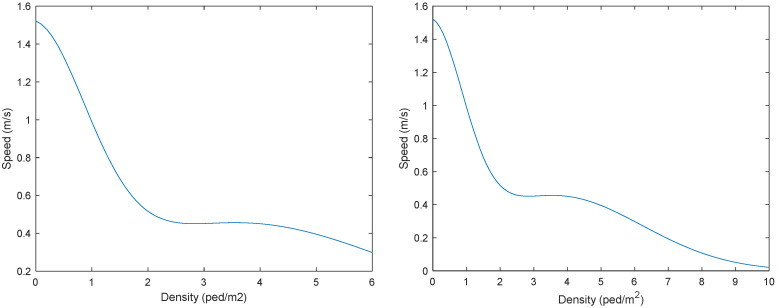
Velocity–density curve.

To accurately describe the basic relationship between pedestrian speed and density under normal conditions, a mathematical model with both high theoretical rationality and good empirical fit needs to be selected. Among numerous speed–density models, the modified Gaussian model proposed by Zhou et al. [16] demonstrates excellent interpretability for observational data due to its ability to accurately depict typical nonlinear characteristics such as the free movement of pedestrians in low-density areas, the peak velocity at the optimal density, and the congestion attenuation in high-density areas. In their research, they reported a relatively low fitting error ($\sigma_{\exp,pred}=3.893\%$). Therefore, this paper adopts this model to simulate the normal evacuation state, and its equation and curve graph are shown in Equation (9) and Figure 11:

(9) $$V\left(\rho \right)=1.371\cdot e^{-{\left(\frac{\rho +0.1524}{1.439}\right)}^{2}}+0.4546\cdot e^{-{\left(\frac{\rho -3.643}{3.653}\right)}^{2}}$$

#### 4.2.2. Panic Value

Panic is a kind of suppressed emotion that people experience when facing threats, extreme pressure or emergencies. It is an instinctive reaction for self-protection when in danger [17]. When subjected to violent attacks, pedestrians may become extremely panicked, which can affect their movement speed, evacuation strategies and evacuation behaviors. Suppose that pedestrians’ panic is mainly influenced by their distance from the exit, their distance from the attacker, and their own personality [18]. When pedestrians approach the attacker or move away from the exit, the level of panic will rise sharply, and those who are sensitive to emotional changes and have a higher emotional response are more likely to panic. Therefore, the panic value is expressed by Equation (10):

(10) $$E_{i}^{ped}\left(t\right)=K\cdot p_{i}\cdot \frac{1-e^{-\beta \min\left(d_{ie}\right)}}{1+e^{-\beta \min\left(d_{it}\right)}}$$

where *K* is the panic coefficient, which is used to control the fluctuation range of panic. *p_i_* is the personality influence factor, a constant random number between 0 and 1 assigned to each pedestrian, representing the sensitivity of pedestrian *i* to panic. *β* is the correction coefficient, making the influence of the distance between the exit and the attacker of the same order of magnitude, with a value of 0.05 [18]; *d_ie_* represents the distance from the pedestrian to the exit. *d_it_* is the distance from a pedestrian to an attacker.

#### 4.2.3. Pedestrian Movement Speed

During emergency evacuation, an individual’s movement speed is closely related to their psychological and physiological state. Scholars have conducted extensive research on this issue, but currently there is no appropriate theory that can comprehensively quantify the influence of these factors. However, research shows that under the stimulation of emergency situations, the average walking speed of pedestrians is significantly higher than that under normal circumstances [19]. Therefore, this model corrects the movement speed of pedestrians based on their own health points and panic values, comprehensively considering the influence of panic values and density on speed. The speed Equation (11) is:

(11) $$V_{i}^{ped}\left(t\right)=r_{i}\times \left(1+E_{i}^{ped}\left(t\right)\right)\times V_{i}^{ped}\left(\rho \right)$$

where *r_i_* represents the loss of life of pedestrian *i*, indicating the degree of injury.

Consider the following two situations:

(1) In a fire situation, *r_i_* represents the decreasing value of the life state of pedestrian *i* over time under the influence of smoke, as shown in Equation (12).

(12) $$r_{i}\left(t\right)=\left\{\begin{array}{l} 1,outside\;the\;smoke\;range\\ e^{-\alpha t},inside\;the\;smoke\;range \end{array}\right.$$

(2) In robbery or earthquake environments, *r_i_* is as shown in Equation (13).

(13) $$r_{i}=\left\{\begin{array}{l} 1,outside\;the\;attack\;range\\ 0,inside\;the\;attack\;range \end{array}\right.$$

#### 4.2.4. Transition Probability

When violent attacks occur, pedestrians usually make choices that are conducive to survival based on the evacuation environment and their own conditions. Meanwhile, the panic of pedestrians can also affect their decision-making process and, in turn, their evacuation strategies. In a bridge or corridor environment, panic will promote the evacuation of pedestrians towards the exit, that is, increase the probability in the (*i*, *j* + 1) direction as shown in Equation (14):

(14) $$P_{i,j+1}=\left(1+E_{i}^{ped}\right)P_{i,j+1}$$

Update the transfer probability in each direction after normalization.

### 4.3. Simulation Setup and Scenario Design

The physical dimensions of the bridge deck were defined as 17.6 m in length and 4 m in width. A discretized grid system was established, with each cell measuring 0.4 m [20]. A computing grid of 44 columns and 10 rows was constructed. When the system is initialized, pedestrian agents are randomly placed at the entrance of the corridor. Each pedestrian occupies a grid cell, and their position status is recorded through a two-dimensional matrix. The simulation core adopts a time-stepping mechanism.

#### 4.3.1. Test Conditions

**Figure 12:**
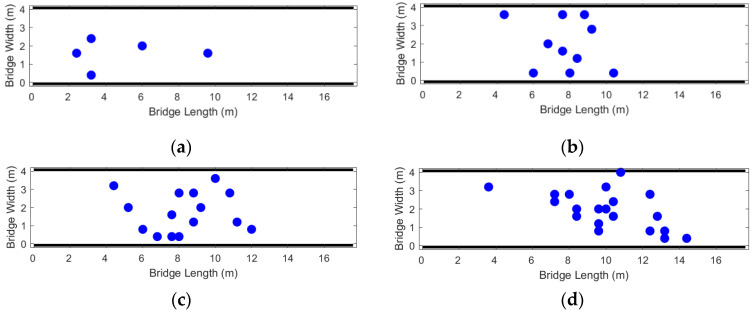
Simulation diagram of each group of working conditions. (**a**) *N* = 5; (**b**) *N* = 10; (**c**) *N* = 15; (**d**) *N* = 20.

**Figure 13:**
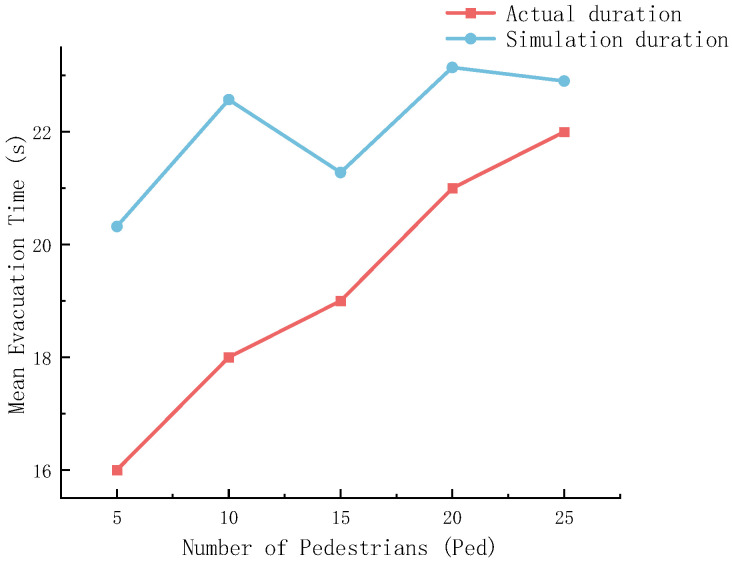
Mean Evacuation Time–Number of Pedestrians.

**Figure 14:**
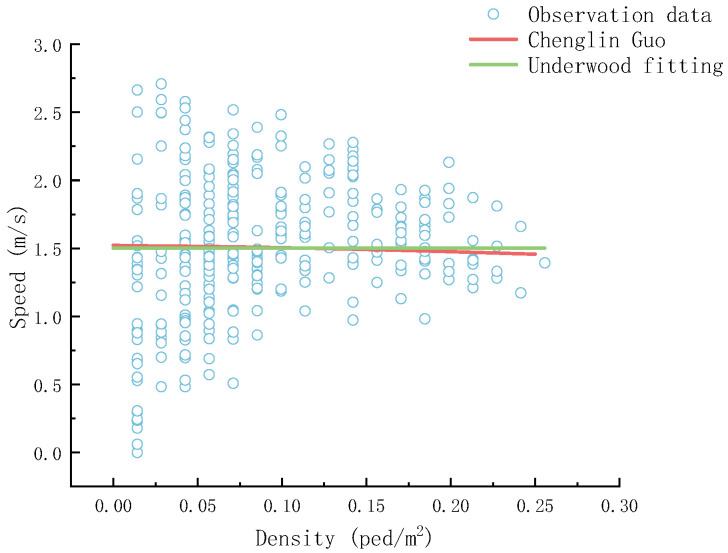
Speed–Density.

The system tracks and records the complete movement trajectory of each pedestrian. When a pedestrian reaches the boundary at the right end of the corridor, it is marked as a successful evacuation, and the evacuation time is recorded. The simulation termination condition is that all pedestrians have completed the evacuation. The evacuation simulation is shown in Figure 12:

To verify the accuracy and reliability of the constructed cellular automaton model for the system, Figure 13 and Figure 14 respectively conduct a comparative analysis of the simulation results with empirical data and classical theoretical models from the two dimensions of evacuation time and velocity–density relationship.

Figure 13 is used to compare the differences between the cellular automaton model and the real scene. The simulated value is greater than the actual value, indicating that the evacuation time is a conservative simulation. Overall, the number of accompanying personnel has increased, and the simulation time has fluctuated upward, which is in line with the actual test situation.

From the overall perspective of Figure 14, with the variation of density, the speed generally shows a relatively stable trend. The Underwood fitting line has well captured the general law of speed variation with density reflected by the observed data, and there is a certain consistency in the trend with the results of Guo [21]. It is indicated that the fitting based on the Underwood model and the observed data in this study, in the research of the speed–density relationship, have similar regular characteristics to existing studies, suggesting that this model has a high degree of fit with the actual situation.

#### 4.3.2. Multi-Group Population

The movement process of a pedestrian group is not a simple superposition or average of individual speeds, but is significantly constrained by the movement correlations among individuals within the group, such as following, avoiding, and coordinating. Among them, the individual with the lowest speed often becomes the key factor restricting the overall advancement efficiency of the group. In this study, the speed of a pedestrian group is clearly defined as the minimum value of the individual pedestrian speed measured among all individual pedestrians that constitute the same pedestrian aggregation group within a specific observation period and spatial range, as shown in Equation (15).

(15) $$V_{k}^{gro}\left(t\right)=\min\left\{V_{1}^{ped}\left(t\right),V_{2}^{ped}\left(t\right),\ldots ,V_{n_{g}}^{ped}\left(t\right)\right\}$$

**Figure 15:**
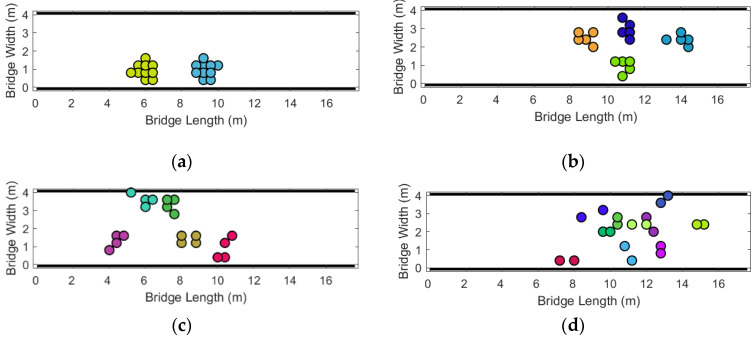
Multi-group evacuation simulation. (**a**) Divide into 2 groups; (**b**) Divide into 4 groups; (**c**) Divide into 5 groups; (**d**) Divide into 10 groups.

**Figure 16:**
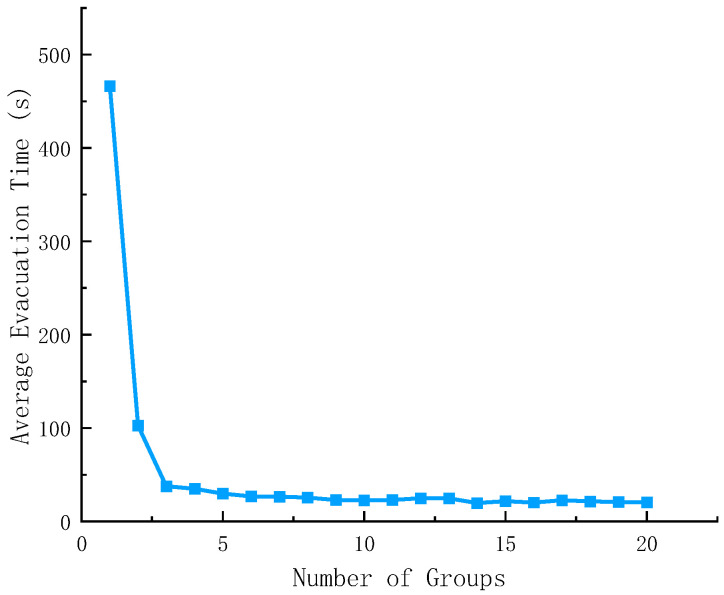
Evacuation time for each group.

**Figure 17:**
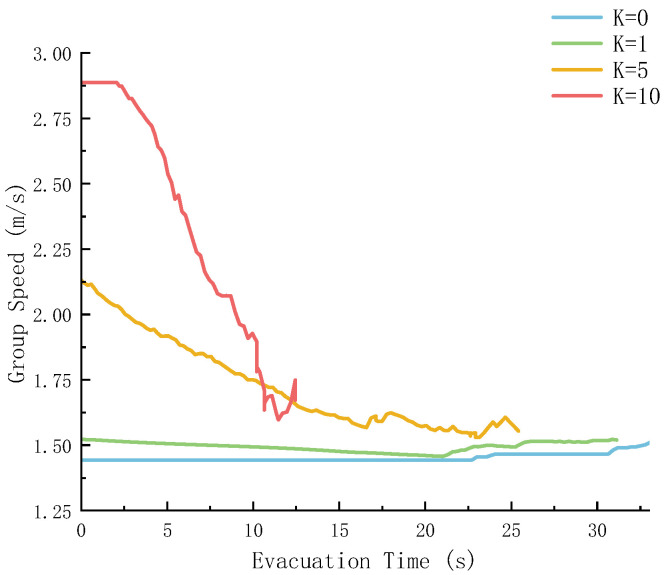
Group velocity under different panic coefficients.

In total, 20 pedestrians were divided into multiple social groups for modeling. Each group has a unique visual identity color, and members maintain a social distance constraint of no more than 0.8 m (2 cells) from each other. The simulation scene is shown in Figure 15:

To explore the combined impact of group structure and panic on evacuation efficiency, this study simulated the evacuation process of multiple groups, and the statistical results are shown in Figure 16 and Figure 17.

Figure 16 shows that when the number of groups is between 0 and 5, the average evacuation time drops sharply as the number of groups increases. When the number of groups is ≥5, the average evacuation time tends to stabilize with a very small decrease.

In Figure 17, as *K* increases, that is, panic intensifies, the initial speed is higher and the attenuation is steeper. This is because panic stimulates the crowd to “rush to escape”, but it also amplifies the impact of congestion, which is consistent with the “non-adaptive acceleration” phenomenon emphasized by Haghani et al. [22] in their review on panic-driven evacuation. The speed difference during the stable stage may be related to the group coordination under panic: when the *N* value is reasonable, such as *N* = 5, it approaches stability in the later stage. Grouping can offset part of the disorder caused by panic and bring the speed back to a stable state [23].

#### 4.3.3. Fire Emergency Situations

Fire scenes are characterized by high danger and irrepeatability. Evacuation involves congestion, avoidance, changes in path selection caused by pedestrians’ panic, as well as the dynamic impact of the fire environment on evacuation decisions.

**Figure 18:**
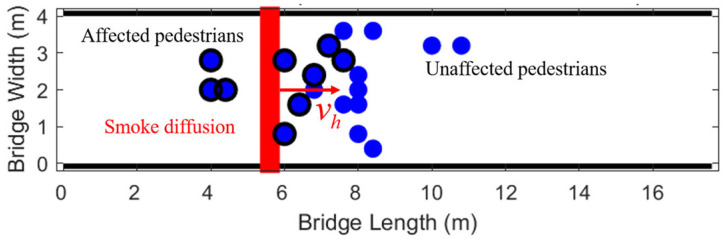
Fire evacuation simulation diagram.

The velocity $v_{h}$ of the hazard source gradually expands over time. In this study, a corresponding cellular automaton simulation visualization system was constructed. The situation at a certain moment is shown in Figure 18.

(1) Set the number of pedestrians *N* = 20, starting from one side of the bridge (L/4), the panic coefficient *K* = 5, the moving speed of the hazard source $v_{h}$ = 0.3 m/s [24], simulating the horizontal diffusion speed v of smoke in the early stage of fire.

**Figure 19:**
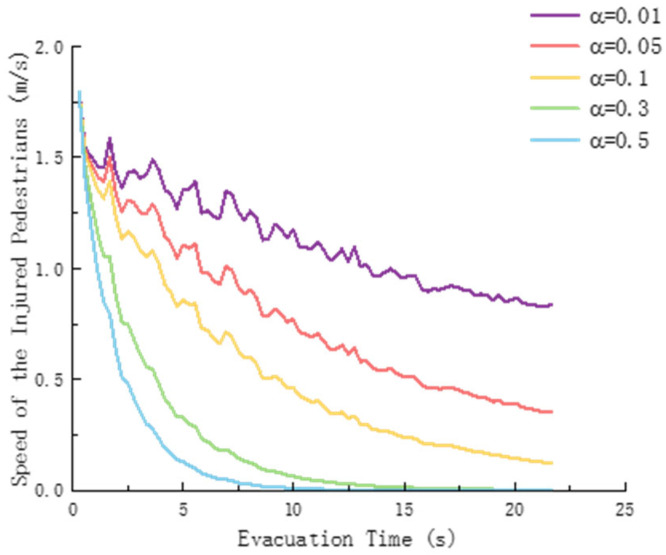
Speed of injured pedestrians.

The rate of life loss α is a key parameter that determines the deterioration rate of the motor ability of injured pedestrians. To quantitatively analyze the dynamic impact of α on the mobility of the injured, Figure 19 depicts the variation curve of the average speed of the injured pedestrian with the evacuation time under different values of α.

Figure 19 shows that as the evacuation time increases, the speed of the injured all shows a downward trend, indicating that the speed of injury during the evacuation process gradually decreases. The larger the *α*, the faster the speed of the injured person decreases. For example, when α = 0.5, the speed drops rapidly to nearly 0. The smaller the α, such as α = 0.01, the slower the speed decrease, the slower the attenuation, and there will still be a certain injury rate after a long time. That is, the greater the exponential decay rate, the more significant the effect on reducing the speed of the injured person, and it can quickly bring the speed of the injured person close to a low level.

(2) The number of pedestrians *N* = 20, starting from one side of the bridge (L/4), the exponential decay rate α = 0.05, the movement speed of the hazard source $v_{h}$ = 0.3 m/s [24].

**Figure 20:**
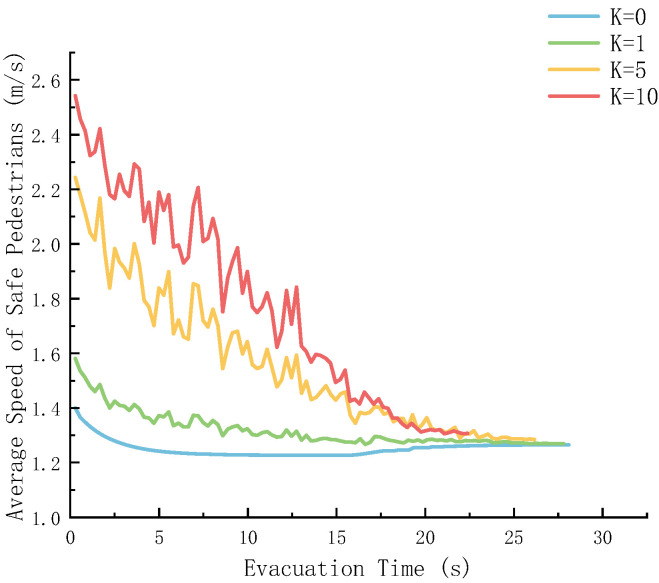
Speed change of safety persons under different panic coefficients.

The panic coefficient *K* directly affects the initial response and subsequent movement state of pedestrians in emergency situations. Figure 20 shows the evolution of the average speed of safe pedestrians with the evacuation time under different levels of panic.

Figure 20 shows that as the evacuation time progresses, the average speed of safe pedestrians at different *K* values all shows a downward trend, indicating that the overall movement speed will gradually decrease and stabilize during the evacuation process. The larger the *K* is, the higher the initial average velocity will be, and the fluctuations during the velocity decline process will also be more obvious.

When *K* = 10, the initial velocity is high, and the velocity fluctuates greatly in the early stage. When *K* = 0, the initial velocity is low, and the decline is gentle, quickly stabilizing. That is, the greater the panic coefficient, the more “rapid” the initial movement of pedestrians, and the more unstable the speed change. Panic will make pedestrians move quickly at the beginning, but it is difficult for them to maintain stability. The panic coefficient is small, pedestrians move more smoothly, and their speed gradually decreases to a stable level.

**Figure 21:**
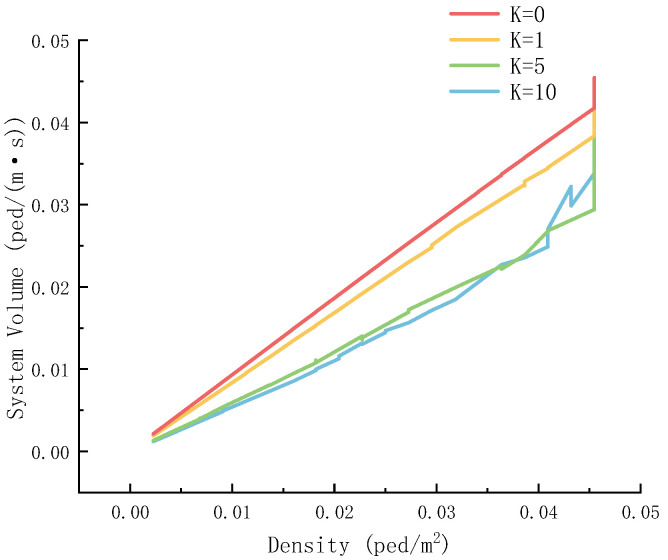
Downstream rate–density diagram of different panic coefficients.

Figure 21 shows the trend of pedestrian flow changing with density under different panic coefficients.

Overall, as the personnel density increases, the system flow rate rises. Moreover, when *K* is different, the growth pattern of flow varies. For instance, when *K* = 0, the growth of flow with density is relatively steeper, reflecting that the impact of personnel density on evacuation flow varies under different degrees of panic.

**Figure 22:**
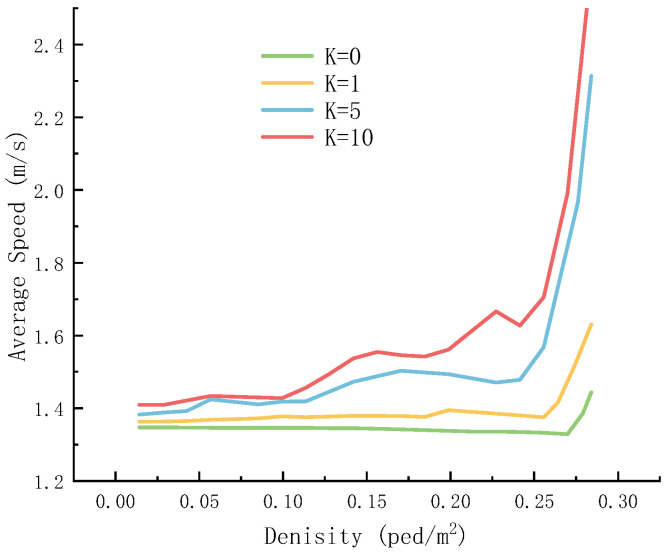
Average velocity–density diagram of the population under different panic coefficients.

Figure 22 further reveals the impact of panic on the relationship between the average speed and density of pedestrians.

Figure 22 shows that the level of panic distorts the relationship between speed and density. Pedestrians exhibit an abnormal acceleration trend at high density. This is because pedestrian movement is affected by the panic value *E* and life injury *r*. At high density, the number of affected pedestrians is small, and panic accelerates the overall speed of the crowd. When the density is low, the damage to life is more severe near the end of the evacuation, and thus the average speed is relatively low, approximately 1.4 m/s.

**Figure 23:**
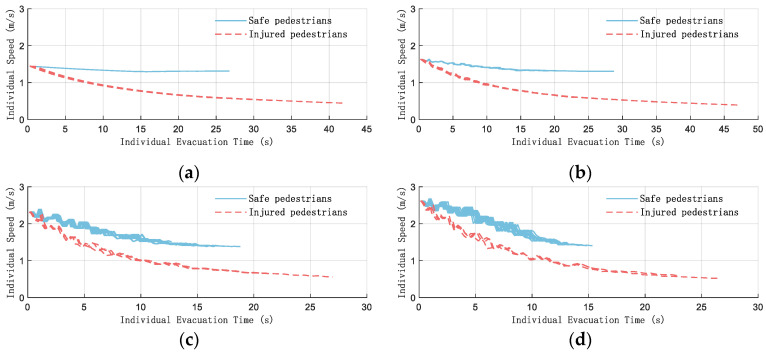
Pedestrian speed during evacuation (N = 20). (**a**) *K* = 0; (**b**) *K* = 1; (**c**) *K* = 5; (**d**) *K* = 10.

The changes in pedestrian speed during the specific simulation evacuation process are shown in Figure 23.

It can be clearly observed from Figure 23 that the loss of life r causes a significant decrease in the speed of pedestrians. As the *K* value increases, the initial pedestrian speed becomes larger, rising from 1.44 m/s to 2.62 m/s, corresponding to the speed of the high-density crowd reflected in Figure 22, which is consistent with the running speed under high panic [21]. The greater the speed fluctuation during the evacuation, the shorter the evacuation time will be.

(3) The number of pedestrians *N* = 40. Starting from a random position on the bridge deck, the exponential decay rate *α* = 0.05, and the panic coefficient *K* = 5.

**Figure 24:**
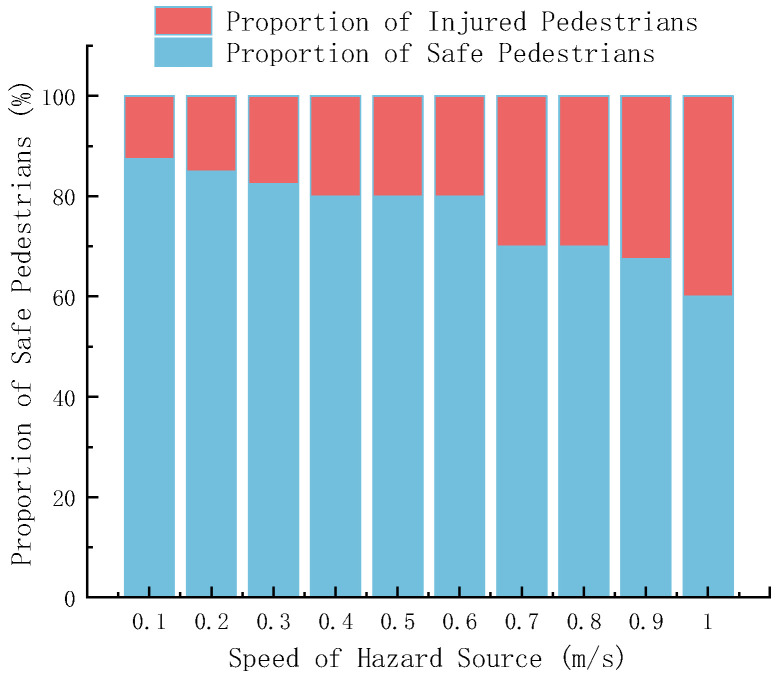
Ratio of safe to injured people under different hazard source speeds.

The movement speed of the hazard source is a key dynamic factor determining the degree of danger in an emergency scene, directly affecting whether pedestrians can successfully evacuate before the threat spreads. Figure 24 reveals the quantitative relationship between the speed of the hazard source and the final safety state of pedestrians.

Figure 24 shows that as the speed of the hazard source increases, the proportion of safe pedestrians generally shows a downward trend, while the proportion of injured pedestrians shows an upward trend. When the speed of the hazard source is 0.1 m/s, the proportion of safe pedestrians is high. When the speed is 1 m/s, the safety ratio decreases and the injury ratio increases, reflecting that the speed of the hazard source has a significant impact on the safety of pedestrians. The greater the speed of the hazard source, the higher the degree of danger faced by pedestrians, the greater the possibility of injury, and the smaller the possibility of safety.

**Figure 25:**
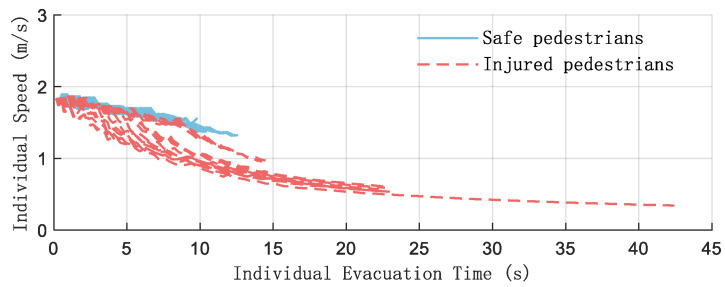
Average speed of injured people under different panic coefficients (*N* = 40, $v_{h}$ = 1 m/s).

Figure 25 clearly shows the speed of each pedestrian during the fire evacuation process when the hazard source $v_{h}$ = 1 m/s.

In the initial stage, 0–5 s, the speed is 1.84 m/s, which conforms to the initial rapid movement characteristics of pedestrian emergency evacuation. In the later stage, after 20 s, the speed tends to level off, and the speed of the injured pedestrian drops below 0.5 m/s. The speed attenuation of the injured pedestrian is more significant, reflecting the setting in the simulation that “injury leads to a decrease in mobility”, which is consistent with the research logic in the evacuation simulation that “personnel status affects behavior”.

#### 4.3.4. Robbery Situation

The evacuation of pedestrians in a robbery scene is not merely a process of spatial movement, but a nonlinear dynamic process interwoven with threat perception, panic, group behavior and evacuation efficiency. On the one hand, the “immediate panic” triggered by the robbery incident can lead to disorder in individual behavior, such as blind running, pushing and shoving, and reverse escape, which in turn can cause secondary risks such as stampedes and congestion. On the other hand, the “visual threat” of weapons can change the path selection of the crowd. Traditional theories based on the assumption of “orderly evacuation”, such as fire and earthquake evacuation models, cannot accurately depict the characteristics of such panic-driven evacuation [25].

**Figure 26:**
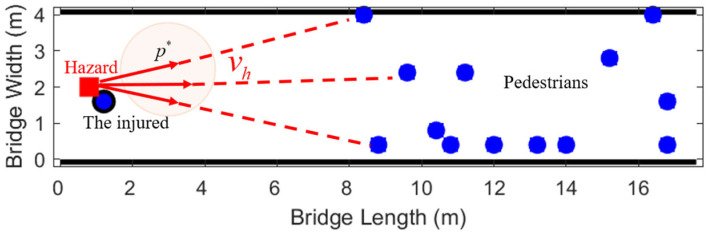
Simulation diagram of robbery evacuation.

To visually simulate the crowd dynamics in the sudden threat scenario of armed robbery, this study constructed the corresponding cellular automaton simulation environment, as shown in Figure 26.

The movement rule of the hazard source in the simulation is shown in Equation (16):

(16) $$\left\{\begin{array}{l} \Delta x_{h}\left(t\right)=v_{h}\cdot \;\Delta t\cdot \frac{x_{p}\left(t\right)-x_{h}\left(t\right)}{\left\|\left.r_{p}\left(t\right)-r_{h}\left(t\right)\right\|\right.}\\ \Delta y_{h}\left(t\right)=v_{h}\cdot \;\Delta t\cdot \frac{y_{p}\left(t\right)-y_{h}\left(t\right)}{\left\|\left.r_{p}\left(t\right)-r_{h}\left(t\right)\right\|\right.} \end{array}\right.$$

where $r_{h}\left(t\right)=\left(x_{h}\left(t\right),y_{h}\left(t\right)\right)$ are the locations of the hazard sources; $r_{p}\left(t\right)=\left(x_{p}\left(t\right),y_{p}\left(t\right)\right)$ is the position of the target pedestrian; $v_{h}$ represents the moving speed of the hazard source; Δt is the time step.

The hazard source targets the injured pedestrian at the smallest distance. The target selection rule is as shown in Equation (17):

(17) $$p^{*}=\arg\min_{p\in P_{\mathrm{active}}}\left\|\left.r_{p}\left(t\right)-r_{h}\left(t\right)\right\|\right.$$

where $P_{active}$ refers to the uninjured gathering of evacuating pedestrians.

**Figure 27:**
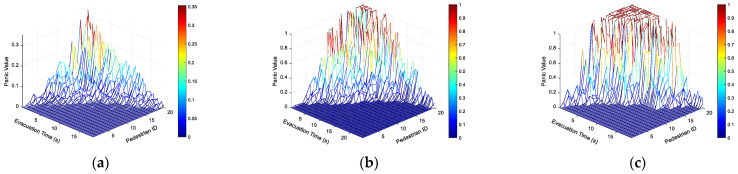
Panic value of pedestrians during evacuation. (**a**) *K* = 1; (**b**) *K* = 5; (**c**) *K* = 10.

**Figure 28:**
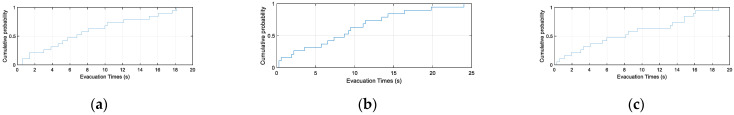
CDF evacuation diagram. (**a**) *K* = 1; (**b**) *K* = 5; (**c**) *K* = 10.

To deeply explore the influence of the panic coefficient *K* on the dynamic evolution of pedestrians’ psychological states during the evacuation process, Figure 27 shows the distribution characteristics of pedestrians’ panic values over time in three typical scenarios: *K* = 1, *K* = 5, and *K* = 10. Figure 28 reveals the cumulative proportion of people who complete evacuation over time under different degrees of panic. A comparison of the three situations reveals significant differential behavioral patterns:

Figure 27 shows that *K* = 1, with a relatively low and gentle peak. The panic coefficient of most pedestrians has not significantly exceeded 0.3, and individual differences are small. Panic rose slowly over time with small fluctuations, and the overall change was stable. When *K* = 5, the peak value increases significantly, with some approaching 1. Moreover, the peak values occur more concentratedly, and the individual panic differentiation intensifies. Panic occurs in the medium term, lasting about 5 to 15 s, with a rapid rise. Later, it remains at a high level or experiences a slight decline. *K* = 10, with an extremely high peak, a large number approaching 1, intense panic, and a long duration of high panic. Panic rapidly reached its peak and then remained at a high level with prolonged fluctuations. Individual differences are extreme. A few pedestrians experience a brief period of low panic followed by a rapid surge or remain at a high level throughout.

Based on Figure 27 and Figure 28, during the evacuation process, on the whole, as time goes by, the number of pedestrians who complete the evacuation-related events keeps accumulating, demonstrating that the evacuation is a gradual process. The longer the time, the higher the proportion of pedestrians achieving the evacuation-related goals, and eventually it approaches full completion. Consistent with the actual data-driven simulation results based on Figure 28 and Wang et al. [26], the effectiveness of this model in simulating the macroscopic evacuation process was further verified.

## 5. Discussion

### 5.1. Research Limitations

Despite the innovative insights and methodological contributions, this study still has certain limitations that need to be addressed in future research:

The experiment was conducted only on an urban overpass with a unidirectional flow design, and the volunteer sample (21 males and 4 females, aged 18–35) lacks diversity in age, gender, and physical condition. The movement characteristics of special groups and the evacuation dynamics in complex spatial structures were not considered, which may limit the generalizability of the model.

The Moore neighborhood transition probabilities were calibrated based on overpass scenario data with pedestrian densities ranging from 0.071 to 0.286 ped/m^2^. The applicability of these parameters in extreme high-density scenarios remains unvalidated. Meanwhile, the hazard source dynamics were simplified to constant speed diffusion or movement, without considering the time-varying characteristics of real hazards.

### 5.2. Future Research Prospects

To address the above limitations and further expand the research depth and application scope, future work will focus on the following directions:

Conduct multi-scenario experiments in public places such as subway stations, stadiums and shopping centers, covering environments with high density (>0.5 people/m^2^), bidirectional flow, multiple exit layouts and dense obstacles. Recruit a more diverse sample, including the elderly, children and the disabled, to collect trajectory data reflecting the movement characteristics of different groups, and optimize the parameter system of the model to achieve cross-scenario adaptation.

Develop a user-friendly pedestrian evacuation simulation platform based on the improved model, providing decision support for the safety design of public buildings and emergency management. Explore the integration of the model with real-time monitoring technologies to realize dynamic prediction and early warning of evacuation risks in actual scenarios.

## 6. Conclusions

This paper takes the urban pedestrian overpass scenario as the research object. By integrating on-site experiments, computer vision analysis and cellular automata modeling, it deeply explores the microscopic movement characteristics and macroscopic evacuation dynamics of pedestrians. The main conclusions are as follows:

1. A pedestrian flow trajectory dataset based on real scenarios was constructed, and YOLO 11 and DeepSORT were utilized to achieve high-precision extraction of pedestrian trajectories and probability statistics of turning angles. For the first time, real turning behaviors were quantified as transition probabilities in the Moore neighborhood of cellular automata, providing data-driven rule calibration for the simulation model.
2. An improved cellular automaton model considering social and psychological factors was developed. The core innovation of the constructed cellular automata evacuation model lies in the introduction of the transfer probability calibration from real data, and comprehensively considers the dynamic influence of key factors such as pedestrian speed–density distribution, panic coefficient *K*, individual life value *rᵢ* and hazard source speed on pedestrian speed.
3. Multi-scenario simulation has revealed the influence mechanisms of key factors such as panic. Through simulations of various scenarios such as regular, group, fire and robbery, a series of valuable conclusions have been drawn:
  (1) The double-edged sword effect of the panic effect. Moderate panic may shorten the evacuation time by enhancing alertness. However, excessive panic can cause behavioral disorder, leading to intensified speed fluctuations. Although it may shorten the evacuation time for some individuals, it will increase the overall risk of congestion and stampedes.
  (2) The constraining effect of group behavior. The number of groups has a significant impact on the average evacuation time, indicating that group movement is constrained by the slowest individual within the group.
  (3) The direct impact of dynamic hazard sources. In fire and robbery scenarios, the increase in the spread or movement speed of hazard sources will directly lead to a decrease in the proportion of safe pedestrians and an increase in the proportion of injured pedestrians, highlighting the importance of controlling hazard sources.

## Figures and Tables

**Table 1:** Experimental settings.

Working Condition Number	Number of Pedestrians (People)	Walking Direction	Repetition Times
Case 1	5	unidirection	4
Case 2	10	unidirection	4
Case 3	15	unidirection	4
Case 4	20	unidirection	4

**Table 2:** Probability of transfer in the Moore neighborhood.

	*N* = 5	*N* = 10	*N* = 15	*N* = 20	*N*
$P\left(i,j-1\right)$	0.094	0.147	0.170	0.163	0.143
$P\left(i+1,j-1\right)$	0.052	0.045	0.035	0.040	0.043
$P\left(i+1,j\right)$	0.020	0.015	0.012	0.018	0.016
$P\left(i+1,j+1\right)$	0.056	0.042	0.044	0.050	0.048
$P\left(i,j+1\right)$	0.674	0.654	0.638	0.629	0.649
$P\left(i-1,j+1\right)$	0.045	0.041	0.050	0.046	0.046
$P\left(i-1,j\right)$	0.018	0.014	0.011	0.016	0.015
$P\left(i-1,j-1\right)$	0.041	0.042	0.040	0.038	0.040

## Data Availability

The original contributions presented in this study are included in the article. Further inquiries can be directed to the corresponding author.
